# Based on Multi-Spectral Technology, the Interaction Mechanism of Xylitol, Stearic Acid and Gelatin Was Explored

**DOI:** 10.3390/foods15213787

**Published:** 2026-10-22

**Authors:** Ligen Wu, Yan Zhang, Anna Wang

**Affiliations:** 1 College of Food Science and Technology, Henan University of Technology, Zhengzhou 450001, China; 2 National Engineering Research Center of Wheat and Corn Further Processing, Henan University of Technology, Zhengzhou 450001, China

**Keywords:** food-grade gelatin modification, non-covalent binding mechanism, multispectral analysis, molecular interaction, fluorescence quenching

## Abstract

To optimize the properties of gelatin-based films and expand their applications in the food industry, this study systematically investigated the interaction mechanisms between xylitol (Xyl), stearic acid (Ste), and gelatin (GA) using multispectral technology combined with thermodynamic analysis and molecular docking simulations. The results demonstrated that both Xyl and Ste formed stable non-covalent complexes with GA through static quenching, and the binding processes are spontaneous. The Xyl-GA complex acts on the amino acid residues of glycine GLY21, alanine ALA20, glutamine GLN19 and arginine ARG17, with a binding constant of 1.35 L/mol and a binding site number of 0.17, and elevated temperature can effectively promote its binding reaction. In contrast, stearic acid–gelatin forms stable complexes with alanine ALA20, valine VAL22, methionine MET23, phenylalanine PHE25 and arginine ARG17 through hydrogen bonds and van der Waals forces. The increase in temperature will weaken the binding force between Ste and GA. Synchronous fluorescence spectroscopy verified that the binding sites of xylitol and stearic acid were adjacent to the tryptophan residues of gelatin. Three-dimensional fluorescence spectroscopy further confirmed that the combination of the two additives can induce conformational changes in the gelatin polypeptide chain. This study characterized the binding sites, characteristic parameters and interaction rules of xylitol/stearic acid and gelatin, and the results may provide partial theoretical guidance for targeted modification of high-performance gelatin-based food packaging materials.

## 1. Introduction

With the increasing global demand for safe and sustainable food packaging, coupled with increasingly stringent environmental regulations on non-degradable plastic waste, the research and development of degradable natural polymer-based food packaging materials and food stabilizers has become a core hotspot in food science and engineering [1]. Gelatin (GA), a natural amphoteric hydrocolloid protein derived from the mild hydrolysis of collagen, exhibits excellent biocompatibility, biodegradability, film-forming ability, and renewability. It can be efficiently extracted from industrial byproducts such as meat processing wastes, thereby enabling resource recycling and aligning with the “dual carbon” development goals. Consequently, GA possesses irreplaceable application value in fields including food packaging, dairy system stability regulation, and food gel product processing, and emerged as one of the research hotspots in natural food polymer materials [2]. However, pure gelatin-based materials suffer from inherent performance limitations. Weak cross-linking between their molecular chains results in insufficient mechanical strength and poor oxygen/water vapor barrier properties of the fabricated films, while the abundance of hydrophilic groups within the gelatin molecule exacerbates these issues. These drawbacks severely restrict the large-scale and diversified applications of gelatin-based materials in the food industry. Consequently, the targeted modification of gelatin to optimize its performance has emerged as a core research direction in this field [3].

Regulating the conformation and intermolecular crosslinking state of gelatin molecules via the incorporation of small-molecule additives is one of the most widely used and technically mature strategies for the modification of gelatin-based materials [4]. Compared with other common modification approaches (including chemical cross-linking, enzymatic modification, and macromolecular composite blending), small-molecule modification has the advantages of simple operation, mild reaction conditions and high biosafety, which is more suitable for the processing requirements of food-grade gelatin materials. Long-chain gelatin molecules possess functional groups (e.g., amino, carboxyl, and hydroxyl groups) capable of interacting with one another, as well as forming non-covalent interactions with small-molecule additives. These interactions alter the molecular aggregation state and microstructure of gelatin, thereby enabling the modulation of its physicochemical properties, including mechanical performance, hydrophobicity, and solubility [5]. Among numerous small-molecule additives for gelatin modification, xylitol (Xyl) and stearic acid (Ste) have garnered extensive attention owing to their distinct structural characteristics and modification advantages. As a natural low-calorie sugar alcohol, Xyl contains multiple hydroxyl groups in its molecular structure, which can form hydrogen bonds or hydrophobic interactions with gelatin molecules. This imparts a plasticizing effect, thereby optimizing the flexibility of gelatin films. Additionally, its excellent biosafety and sweetness-modulating properties endow it with unique advantages for the modification of food-grade gelatin-based materials. In contrast, stearic acid, a typical hydrophobic saturated fatty acid, features long-chain hydrophobic alkyl groups in its molecule. These groups can bind to the hydrophobic domains of gelatin, effectively enhancing the hydrophobicity and moisture resistance of gelatin-based materials [6]. Meanwhile, it can modulate the crosslinking state of gelatin molecules via hydrogen bonding and van der Waals forces, thereby enhancing the barrier properties of the resulting materials [7]. They represent hydrophilic and hydrophobic small-molecule additives, respectively. Theoretically, the molecular conformation and microstructure of gelatin can be synergistically modulated from both hydrophilic and hydrophobic dimensions, enabling the comprehensive optimization of gelatin-based material properties. Consequently, the regulatory mechanism of the composite system comprising xylitol, stearic acid, and gelatin emerges as a critical research focus in the field of food colloidal materials [8].

To date, the interactions between gelatin and individual small-molecule additives have been preliminarily investigated. Specifically, existing studies have confirmed that xylitol modulates the gel properties of gelatin, while stearic acid enhances the hydrophobicity of gelatin films [9]. However, existing studies still have notable limitations, and a systematic theoretical framework for the interaction between gelatin and small-molecule additives with different polarities has not been fully established, making it challenging to guide the precise modification of gelatin-based materials [9]. First, existing research has focused primarily on the interactions between gelatin and individual additives, lacking a systematic comparative analysis of the interaction mechanisms between gelatin and two small molecules of distinct polarities—xylitol and stearic acid. Specifically, the binding specificity of these two molecules to gelatin, differences in the types of intermolecular forces involved, and variations in their binding sites have not been clearly elucidated. As a result, no solid theoretical basis has been established to support the composite modification of gelatin using these two additives [10]. Second, the research methodologies employed are relatively simplistic, relying primarily on a single spectroscopic technique for characterization. Consequently, it remains challenging to reveal the conformational changes in gelatin at the molecular level during its interaction with small molecules, and the microscopic mechanisms underlying these interactions have not been comprehensively elucidated [11]. Third, temperature fluctuations during food processing can alter the interactions between gelatin, xylitol, and stearic acid. However, the regulatory effects of temperature on the binding affinity and binding modes of xylitol and stearic acid to gelatin have not yet been elucidated in existing research. Consequently, gelatin modification in practical production remains limited to empirical regulation, with no capacity for targeted optimization tailored to specific processing technologies [12]. These aforementioned challenges emerge as a key bottleneck impeding the performance breakthroughs and industrial-scale application of gelatin-based materials. Consequently, an in-depth, systematic investigation into the interaction mechanisms among xylitol, stearic acid, and gelatin holds considerable academic value and profound practical application significance.

Against this backdrop, gelatin, xylitol, and stearic acid were selected as the research subjects for this study. Multispectral techniques, combined with thermodynamic analysis and molecular docking simulation approaches, were employed to systematically investigate the interaction mechanisms among xylitol, stearic acid, and gelatin. Specific parameters—including the type of fluorescence quenching, binding constant, and number of binding sites—for the interactions between each of the two compounds and gelatin were determined. The primary types of intermolecular forces and thermodynamic characteristics governing the binding of xylitol and stearic acid to gelatin were analyzed, and the specific binding sites and modes of these two molecules on gelatin chains were identified. Meanwhile, the regulatory effects of temperature variation on the xylitol-gelatin and stearic acid-gelatin interactions were explored, and the distinct differences in the binding behaviors between gelatin and these two small molecules of contrasting polarities were elucidated. Compared with existing relevant studies, the core innovations of this work are reflected in the following three aspects: (1) Systematic comparative analysis of the interaction differences between gelatin and two small molecules with significant polarity differences (hydrophilic xylitol and hydrophobic stearic acid), clarifying the heterogeneity of their binding specificity and action modes; (2) Revealing the regulatory effect of food processing-related temperature changes on the binding affinity of the two systems, which provides a basis for process parameter regulation in practical production; (3) Adopting multi-technique combined analysis to overcome the limitations of single characterization methods, and comprehensively elucidating the microscopic interaction mechanism at the molecular level. This work aims to address the existing research gaps in the interaction mechanisms between gelatin and small-molecule additives, refine the theoretical framework underpinning gelatin-small molecule additive interactions, and provide critical theoretical support and a scientific basis for the precise modification of gelatin-based materials. In turn, these findings are expected to facilitate the development of high-performance food-grade gelatin-based packaging materials and food stabilizers, thereby promoting the expanded application and industrialization of gelatin within the food industry.

## 2. Materials and Methods

### 2.1. Materials and Chemicals

Gelatin (analytical grade) was purchased from Shanghai Komio Chemical Reagent Co., Ltd. (Shanghai, China). Xylitol (analytical grade) was obtained from Shandong Futian Pharmaceutical Co., Ltd. (Dezhou, China). Stearic acid (analytical grade) was supplied by Shanghai Macklin Biochemical Technology Co., Ltd. (Shanghai, China). Anhydrous ethanol (analytical grade) was purchased from Tianjin Kemiou Chemical Reagent Co., Ltd. (Tianjin, China). Hydrochloric acid (analytical grade) was obtained from Shanghai Aladdin Biochemical Technology Co., Ltd. (Shanghai, China). Sodium hydroxide (analytical grade) was supplied by Tianjin Zhiyuan Chemical Reagent Co., Ltd. (Tianjin, China).

### 2.2. Preparation of Sample Solutions

The preparation methods were based on the method of Moon and Kweon [13] with the following specific modifications: (1) The mass concentration of gelatin stock solution was adjusted to 2% (*w*/*v*) to meet the test requirements of subsequent multi-spectral analysis; (2) The dissolution condition was optimized to 60 °C water bath with magnetic stirring, which can ensure the complete dissolution of gelatin while avoiding protein denaturation caused by excessive temperature.

A 2% (*w*/*v*) gelatin solution was prepared as follows: 2.00 g of gelatin was accurately weighed and dispersed in 100 mL of deionized water. The mixture was heated in a 60 °C water bath and magnetically stirred until the gelatin was completely dissolved, yielding a 2% (*w*/*v*) gelatin stock solution. The stock solution was then cooled to room temperature and stored for subsequent use.

For the 0.0562 mol/L stearic acid stock solution, 1.60 g of stearic acid was accurately weighed and transferred into a 50 mL beaker. Five milliliters (5 mL) of anhydrous ethanol was added to the beaker, and the mixture was heated to 70 °C to achieve complete dissolution. After full dissolution, deionized water was slowly added dropwise, and the solution was diluted to a final volume of 100 mL. The prepared 0.0562 mol/L stearic acid stock solution was then stored for subsequent use.

Xylitol solution: 0.76 g, 1.52 g, 3.04 g, 4.56 g, 6.09 g, 7.61 g were accurately weighed into 6 beakers, respectively, and 100 mL of deionized water was added to each beaker. The mixtures were placed in a magnetic stirring water bath at 60 °C until the xylitol was completely dissolved. The sugar solution in the 6 beakers was transferred to a 100 mL volumetric flask and diluted to 100 mL with deionized water to obtain 0.05 mol/L, 0.1 mol/L, 0.2 mol/L, 0.3 mol/L, 0.4 mol/L, and 0.5 mol/L xylitol solutions. The solutions were then mixed thoroughly at room temperature.

### 2.3. Preparation of Interaction Sample Solution

Preparation of xylitol-gelatin (Xyl-GA) interaction solution: 2 mL GA solution (2% *w*/*v*) was transferred into a centrifuge tube, and 1 mL Xyl solution (concentration of 0.05, 0.1, 0.2, 0.3, 0.4, 0.5 mol/L) was added, and the mixture was vortexed for 30 s. The final mass concentration of GA in the Xyl-GA interaction system was 1.33% (*w*/*v*), and the final concentrations of Xyl were 0.017, 0.033, 0.067, 0.100, 0.133 and 0.167 mol/L, respectively. The centrifuge tube was placed in a constant temperature water bath at 298 K and 310 K for 20 min, and the fluorescence spectrum was measured.

The configuration ratio of stearic acid-gelatin (Ste-GA) interaction solution is shown in Table 1. According to the ratio of Table 1, the corresponding volume of gelatin solution, stearic acid solution and deionized water was pipetted into a centrifuge tube, and magnetically stirred at room temperature for 2 h to fully dissolve it. The mixture was hydrated statically at 4 °C overnight, allowed to return to room temperature, and the pH of the solution was adjusted to 7.0 using 0.1 mol/L hydrochloric acid and 0.1 mol/L sodium hydroxide solution for subsequent measurements.

### 2.4. Ultraviolet Spectrometric Determination

At room temperature, the ultraviolet spectra of the mixed samples were scanned using an ultraviolet-visible spectrophotometer (UV-3400S, Shanghai, China). First, Xyl solution (concentration gradient of 0.05, 0.1, 0.2, 0.3, 0.4, 0.5 mol/L) and Ste solution (concentration gradient of 0, 2.81 × 10^−3^, 5.62 × 10^−3^,11.24 × 10^−3^, 16.86 × 10^−3^, 22.48 × 10^−3^ mol/L) were added to the GA stock solution at a ratio of 1:1, and then placed on a vortex oscillator for 1 min to ensure that the components were fully mixed. The 1:1 mixing ratio was specially set for UV measurement to ensure that the absorbance of the sample was within the optimal linear range of the instrument, avoiding test deviation caused by too high or too low absorbance, which is a conventional optimization for UV spectral detection. After incubation at room temperature for 10 min, the mixed samples were scanned by ultraviolet spectroscopy with a scanning wavelength range of 190–400 nm. Deionized water was used as the control group, and the spectral scanning conditions were set according to the method of Zao [14].

### 2.5. Determination of Fluorescence Spectra

The endogenous fluorescence spectrum analysis was carried out according to the method of Yuan [15]. The instrument used was a fluorescence spectrophotometer (Cary Eclipse, Agilent, Santa Clara, CA, USA). The specific working parameters of the instrument were set as follows: excitation wavelength of 280 nm, gain photomultiplier tube of 350 V, excitation and emission bandwidth of 10 nm, scanning speed of 1000 nm/min, scanning range of 200–600 nm, and emission slit width of 5 nm.

### 2.6. Calculation of Quenching Type

Referring to the method of Astolfi [16], the Stern-Volmer equation was used to determine the fluorescence quenching mechanism of xylitol and stearic acid on gelatin.

(1) $$\frac{F_{0}}{F}=1+K_{q}\tau_{0}\left[Q\right]=1+K_{\mathrm{SV}}\left[Q\right]$$

In the formula, F_0_ represents the endogenous fluorescence of gelatin; F represents the endogenous fluorescence of gelatin after binding to xylitol and stearic acid; K_q_ represents the quenching rate constant; τ_0_ indicates the fluorescence lifetime of the protein (10^−8^ s for gelatin); K_SV_ represents the Stern-Volmer quenching constant.

### 2.7. Calculation of Binding Constants and Binding Sites

According to the method of Masaeli [17], the binding constants and the number of binding sites of xylitol and stearic acid to gelatin were calculated by double logarithmic equation.

(2) $$\mathrm{lg}\frac{F_{0}-\;F}{F}=\mathrm{lg}K_{a}+\mathrm{nlg}\left[Q\right]$$

In the formula, K_a_ is the binding constant; n denotes the number of binding sites.

### 2.8. Calculation of Thermodynamic Parameters and Acting Force

Referring to the method of Bente [18], the changes in thermodynamic parameters (enthalpy change, entropy change and Gibbs free energy) were calculated by using Van’t Hoff equation and thermodynamic equation. According to the calculated thermodynamic parameters, the reaction driving force between xylitol and stearic acid on the interaction between gelatin was speculated.

(3) $$\mathrm{lnK}=-\frac{∆H}{\mathrm{RT}\;}+\frac{∆S}{R}$$

(4) ∆G=∆H − T∆S

In the formula, K represents the binding constant; T denotes the absolute temperature; R represents the gas constant, 8.314 J·mol^−1^K^−1^; ∆H represents the enthalpy change; ∆S denotes entropy change; ∆G denotes the change in Gibbs free energy. According to the size and positive and negative of the parameters, the type of interaction force can be judged: when ∆H < 0, ∆S > 0, the force is electrostatic interaction; when ∆H > 0, ∆S > 0, the force is hydrophobic interaction; when ∆H < 0, ∆S < 0, the forces are hydrogen bond and van der Waals force [19].

### 2.9. Synchronous Fluorescence Spectrometric Determination

According to the method of [20], synchronous fluorescence can reduce the influence of scattered light on the spectrum, and has high selectivity and sensitivity. When the wavelength scanning range is 200–600 nm, the synchronous fluorescence spectra of the mixed system samples at ∆λ = 15 nm and ∆λ = 60 nm were measured.

### 2.10. Determination of Three-Dimensional Fluorescence Spectroscopy

The three-dimensional fluorescence spectra of the samples were recorded at room temperature by referring to the method of [21]. The spectral recording range was 200–600 nm and gradually increases with 10 nm, and the scanning rate is 10,000 nm/min. The other conditions were the same as those of fluorescence spectrometry.

### 2.11. Molecular Docking Simulation

Molecular docking was performed via the CDOCKER method using Discovery Studio 2019 Client software. The three-dimensional (3D) structure of GA (PDB ID: 5cvb) was retrieved from the RCSB PDB protein database. This structure was selected because it is a high-resolution (1.8 Å) crystal structure of collagen-like peptide, which is the core structural unit of gelatin, and has been widely used in the simulation study of gelatin-small molecule interaction [22]. The two-dimensional (2D) structures of Xyl (CID: 6420179) and Ste (CID: 121860) were obtained from the PubChem database. Before docking, the potential binding pockets of GA were predicted and analyzed using the “Define and Edit Binding Site” module in the software, and a total of 5 potential binding pockets were identified. All potential binding sites were considered during the docking analysis. Subsequently, the structures of Xyl, Ste, and GA were imported into Discovery Studio 2019 Client, and the protein structure was pretreated by removing water molecules and adding hydrogen atoms. The CHARMm force field was used for structural optimization of ligands and receptor proteins. The key docking parameters were set as follows: the docking sphere was set with a radius of 15 Å centered on the potential binding pocket, the top 10 conformations were retained for each ligand, the number of random conformations was set to 10, and other parameters adopted the default settings of the software. Based on the interaction energy values, the optimal docking conformations between gelatin (GA), xylitol (Xyl), and stearic acid (Ste) were identified, and the docking interaction results were systematically analyzed.

### 2.12. Statistical Analysis

All experimental data are expressed as mean ± standard deviation (SD) of three independent experiments. Before statistical analysis, the normality of data distribution was verified by Shapiro–Wilk test, and the homogeneity of variance was verified by Levene’s test. The SPSS software program (version 16.0, SPSS Inc., Chicago, IL, USA) was used to perform one-way analysis of variance (ANOVA) on all data, and Duncan’s multiple range test was used for post hoc testing, *p* < 0.05 was statistically significant. Origin 2021 was used for software drawing.

## 3. Results and Discussion

### 3.1. Ultraviolet Spectrum Analysis

The interaction of GA with Xyl and Ste can be evaluated by analyzing the UV absorption spectra [23]. As shown in Figure 1, the UV-visible spectra of the experimental samples were scanned over the wavelength range of 200–400 nm. A characteristic absorption peak of GA was observed in the range of 200–220 nm, which is mainly attributed to the aromatic amino acid residues contained in its molecular structure, including tryptophan (Trp), tyrosine (Tyr), and phenylalanine (Phe) [24]. With an increase in Xyl concentration, a significant increase in the absorbance of the Xyl-GA system was observed, accompanied by a notable blue shift in the maximum absorption wavelength-specifically, the maximum absorption peak was blue-shifted from 220 nm to 210 nm. This characteristic blue shift phenomenon indicated that the interaction between Xyl and GA changed the microenvironment of the chromophore in GA molecules, which may be related to the formation of non-covalent interactions between the two components [25]. This phenomenon also indicates that hydrophobic interaction serves as the main driving force for the interaction between Xyl and GA.

### 3.2. Fluorescence Spectrum Analysis

The endogenous fluorescence of gelatin is mainly derived from the benzene ring structure of tyrosine and tryptophan residues in its molecule [26]. Specifically, these aromatic amino acid residues, which were previously identified as key components of GA, are responsible for the intrinsic fluorescent properties of gelatin. A characteristic fluorescence emission peak is exhibited at approximately 340 nm, and the spectral response in this region can directly reflect the polarity, hydrophobicity, and spatial conformational changes in the microenvironment surrounding the aromatic amino acid residues following the binding of small-molecule ligands to gelatin [27]. Protein quenching induced by small molecules occurs through three primary mechanisms: static quenching (formation of ground-state complexes), dynamic quenching (molecular collisions), and a combination of static and dynamic quenching [28]. In this study, the interaction mechanisms between Xyl, Ste, and GA were investigated via fluorescence quenching spectroscopy at 298 K and 310 K. As shown in Figure 2, with the increase in Xyl concentration, the fluorescence intensity at the characteristic emission peak of GA (around 340 nm) showed a gradual quenching trend, while the fluorescence intensity of the system at the same Xyl concentration decreased with the increase in temperature from 298 K to 310 K [29]. This phenomenon can be attributed to the fact that the binding of Xyl to GA induces the exposure of more Trp and Tyr residues originally buried in the hydrophobic core, leading to the enhancement of the overall fluorescence signal, which is consistent with the subsequent analysis of synchronous fluorescence and three-dimensional fluorescence results. In contrast, in the Ste-GA system, no significant increase in the degree of fluorescence quenching was observed with increasing temperature. This phenomenon is thought to result from the formation of hydrogen bonds between Ste and the amide groups surrounding the aromatic amino acid residues in GA, or from weak van der Waals interactions between Ste and gelatin [30]. This mild non-covalent binding only slightly alters the microenvironment of the fluorescent chromophores, leading to a slight reduction in fluorescence emission efficiency. Since temperature exerts a weak effect on hydrogen bonding, the fluorescence emission process is effectively suppressed [31].

### 3.3. Judgment of Quenching Type, Binding Site and Binding Constant

As shown in Figure 3, at 298 K and 310 K, a good linear upward trend in the F_0_/F values of both the Xyl-GA and Ste-GA systems was observed with increasing ligand concentration [Q]. The two fitted lines exhibited high linear correlation, indicating that the fluorescence quenching of gelatin by xylitol and stearic acid was consistent with the linear characteristics of the Stern-Volmer equation [32]. Experimental results demonstrated that a downward trend in the quenching constants of both the Xyl-GA and Ste-GA systems was observed with increasing temperature, indicating that the quenching process of GA by Xyl and Ste was dominated by a static quenching mechanism, which was further verified by the temperature-dependent quenching constant changes. The binding constant (ka) is an important parameter used to evaluate the strength of protein-ligand interactions; a higher ka value indicates a stronger binding ability between the two, while a lower value indicates a weaker binding ability [33]. The ka values and the number of binding sites (n) for the Xyl-GA and Ste-GA systems at different temperatures were calculated using double logarithmic equations, with the results presented in Table 2. A significant increase in the binding constants of both the Xyl-GA and Ste-GA systems was observed with increasing temperature, and the number of binding sites exhibited a consistent trend. This result is consistent with the aforementioned analysis: an increase in temperature induces conformational changes in GA, which exposes more fluorophores to the solvent environment and thereby increases the number of binding sites for Xyl and Ste on GA [34].

### 3.4. Thermodynamic Parameters and Force Analysis

According to thermodynamic theory, if ΔH > 0 and ΔS > 0, hydrophobic interaction is indicated to play a dominant role in the binding of the two substances. If ΔH < 0 and ΔS > 0, electrostatic interaction is suggested to be the main driving force. In contrast, ΔH < 0 and ΔS < 0 imply that van der Waals forces or hydrogen bonds may act as the primary forces [35]. Table 3 demonstrated that hydrophobic interaction plays a major role in the formation of the Xyl-GA complex, while hydrogen bonds and van der Waals forces dominate the Ste-GA system. Additionally, the ΔG values of all systems were negative at both test temperatures, indicating that the binding processes between Xyl/Ste and GA were spontaneous. The sign of enthalpy change (ΔH) determines the exothermic or endothermic nature of the reaction: the ΔH of the Xyl-GA system was positive, indicating that the binding process was endothermic; while the ΔH of the Ste-GA system was negative, indicating that the binding process was exothermic [36]. A larger negative ΔG value indicates a more facile reaction [37]. With increasing temperature, a gradual decrease in the ΔG value of the Xyl-GA system was observed, whereas an upward trend in the ΔG value was noted for the Ste-GA system. This phenomenon indicates that an increase in temperature is favorable for the reaction in the Xyl-GA system, and hydrophobic interaction may significantly enhance the stability of the complex [38]. In contrast, the increase in the ΔG value of the Ste-GA system with rising temperature suggests that hydrogen bonds and van der Waals forces are more temperature-sensitive, and elevated temperatures may weaken the binding capacity of these systems [39].

### 3.5. Synchronous Fluorescence Spectra

Synchronous fluorescence spectroscopy exhibits excellent sensitivity and specificity in qualitative and quantitative analysis, with the ability to effectively alleviate spectral contour overlap and suppress light-scattering interference [40]. Changes in the microenvironment surrounding specific amino acids can be identified by alterations in synchronous fluorescence peaks, where the synchronous fluorescence peaks of Tyr and Trp correspond to wavelength intervals of 15 nm and 60 nm, respectively.

In this study, two characteristic wavelength intervals (∆λ = 15 nm and ∆λ = 60 nm) were selected to investigate the differential effects of Xyl and Ste on the microenvironments of Tyr and Trp in GA. Figure 4 showed that the fluorescence intensity and degree of quenching of the system under the condition of ∆λ = 60 nm were significantly higher than those under ∆λ = 15 nm, indicating that the interaction between Xyl/Ste and GA had a more significant effect on the microenvironment of Trp residues, and the binding sites of the two small molecules to GA may be closer to Trp residues [41]. Additionally, a significant decrease in the fluorescence intensity of GA was observed with increasing concentrations of Xyl and Ste, demonstrating that Xyl and Ste can effectively quench the endogenous fluorescence of GA—consistent with the results of previous studies.

### 3.6. Three-Dimensional Fluorescence Spectra

To further explore the conformational and microenvironmental changes in GA in the presence of Xyl and Ste, three-dimensional fluorescence spectroscopy was employed for systematic analysis. The three-dimensional fluorescence spectra of the Xyl-GA (a) and Ste-GA (b) systems are presented in Figure 5. Among these spectra, Peak a and Peak b correspond to the first-order Rayleigh scattering peak (λem = λex) and the second-order Rayleigh scattering peak (λem = 2λex), respectively [42]. Peak 1 is located at an excitation wavelength of 280 nm and primarily reflects the fluorescence characteristics of tryptophan (Trp) and tyrosine (Tyr) residues in GA, while Peak 2 is associated with the fluorescence characteristics of the GA polypeptide chain backbone [43]. With increasing concentrations of Xyl and Ste, a significant downward trend in the fluorescence intensity of Peak 1 was observed for both the Xyl-GA and Ste-GA complexes, and the fluorescence intensity of Peak 2 for these complexes decreased significantly or even disappeared completely. These phenomena indicated that the interaction between Xyl/Ste and GA changed the microenvironment of the fluorescent chromophore in GA, which was related to the alteration of amino acid residue polarity and the conformational change in GA polypeptide chain [44]. The results provided indirect evidence for the formation of Xyl-GA and Ste-GA complexes, which was consistent with the conclusions of the above fluorescence spectrum and thermodynamic analysis [45].

### 3.7. Molecular Docking Simulation Results

As shown in Figure 6a, xylitol interacts with glycine (GLY21), alanine (ALA20), glutamine (GLN19) and arginine (ARG17) residues on gelatin molecules. The docking results of stearic acid and gelatin showed that the binding sites were located near the residues of alanine (ALA20), valine (VAL22), methionine (MET23), phenylalanine (PHE25) and arginine (ARG17) of gelatin molecules.

In contrast, Figure 6b demonstrates that stearic acid does not bind to the polar surface of gelatin but is deeply embedded in a compact nonpolar core domain of the gelatin molecule, located at the junction of the red α-helix and the gray folded backbone region, with binding sites concentrated around alanine (ALA20), methionine (MET23), valine (VAL22), and phenylalanine (PHE25) residues [46]. This binding pocket is characterized by a dense arrangement of nonpolar amino acid side chains, including tryptophan (Trp) and tyrosine (Tyr), creating a nonpolar microenvironment that favors accommodation of the long aliphatic chain of stearic acid, which is highly consistent with the previous conclusion from synchronous fluorescence spectroscopy that stearic acid acts on Trp residues; this stands in sharp contrast to the binding mode of xylitol to the polar surface, directly explaining the differences in the intensity and site specificity of the two interactions [47].

In summary, the binding of Xyl and Ste to GA primarily relies on van der Waals forces, hydrogen bonds, and hydrophobic interactions. This conclusion is highly consistent with the results of thermodynamic analysis, which further confirms the stability and binding mechanisms of the Xyl-GA and Ste-GA complexes.

## 4. Conclusions

Multispectral technology combined with thermodynamic analysis and molecular docking simulation was employed to explore the interaction mechanisms of xylitol and stearic acid with gelatin. Both xylitol and stearic acid spontaneously formed stable non covalent complexes with gelatin via static dominated fluorescence quenching. The xylitol–gelatin interaction, dominated by hydrophobic forces, was endothermic and promoted by temperature elevation, with xylitol interacting with GLY21, ALA20, GLN19 and ARG17 residues. Stearic acid embedded into the hydrophobic core of gelatin and bound to ALA20, VAL22, MET23, PHE25 and ARG17 residues through hydrogen bonds and van der Waals forces; this exothermic binding was weakened upon a temperature increase. Synchronous fluorescence indicated that the binding sites of both compounds were close to gelatin tryptophan residues. Three dimensional fluorescence further demonstrated that gelatin polypeptide conformation changed after binding with xylitol or stearic acid. This study clarifies the binding parameters and binding site information of the two small molecule additives toward gelatin, offering critical theoretical support for the precise modification of gelatin based food materials.

## Figures and Tables

**Figure 1:**
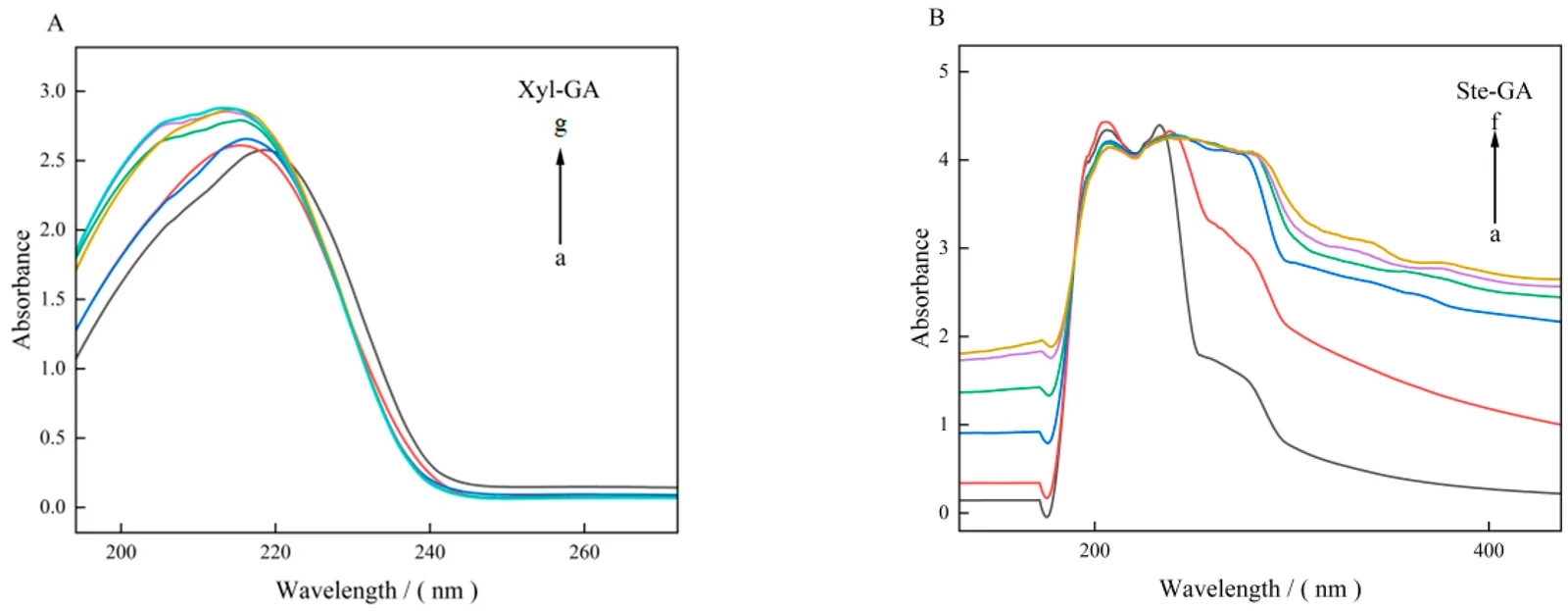
UV absorption spectra of the interaction of Xyl (**A**), Ste (**B**) with GA. Note: (**A**) curve a–g corresponding to the concentration of xylitol added was 0, 0.05, 0.1, 0.2, 0.3, 0.4, 0.5 mol/L; (**B**) curve a–f corresponded to stearic acid concentration of 0, 2.81 × 10^−3^, 5.62 × 10^−3^, 11.24 × 10^−3^, 16.86 × 10^−3^, 22.48 × 10^−3^ mol/L.

**Figure 2:**
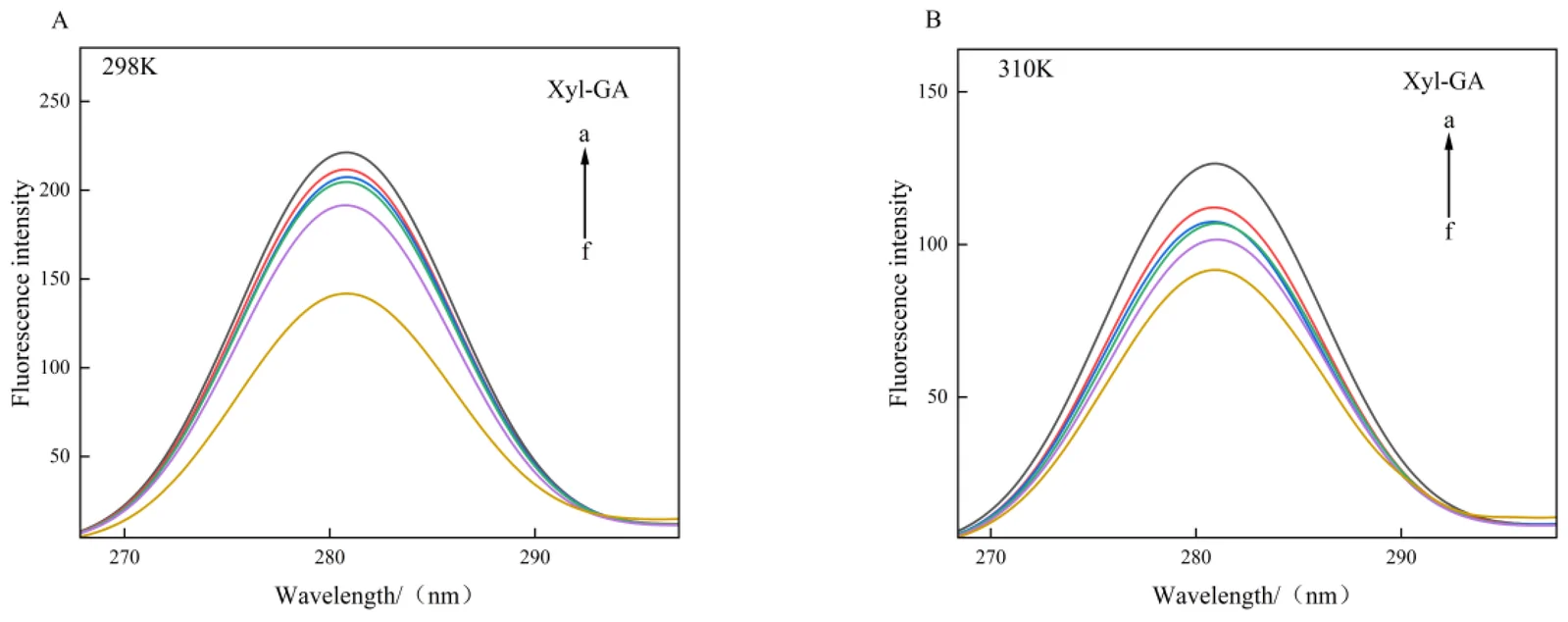

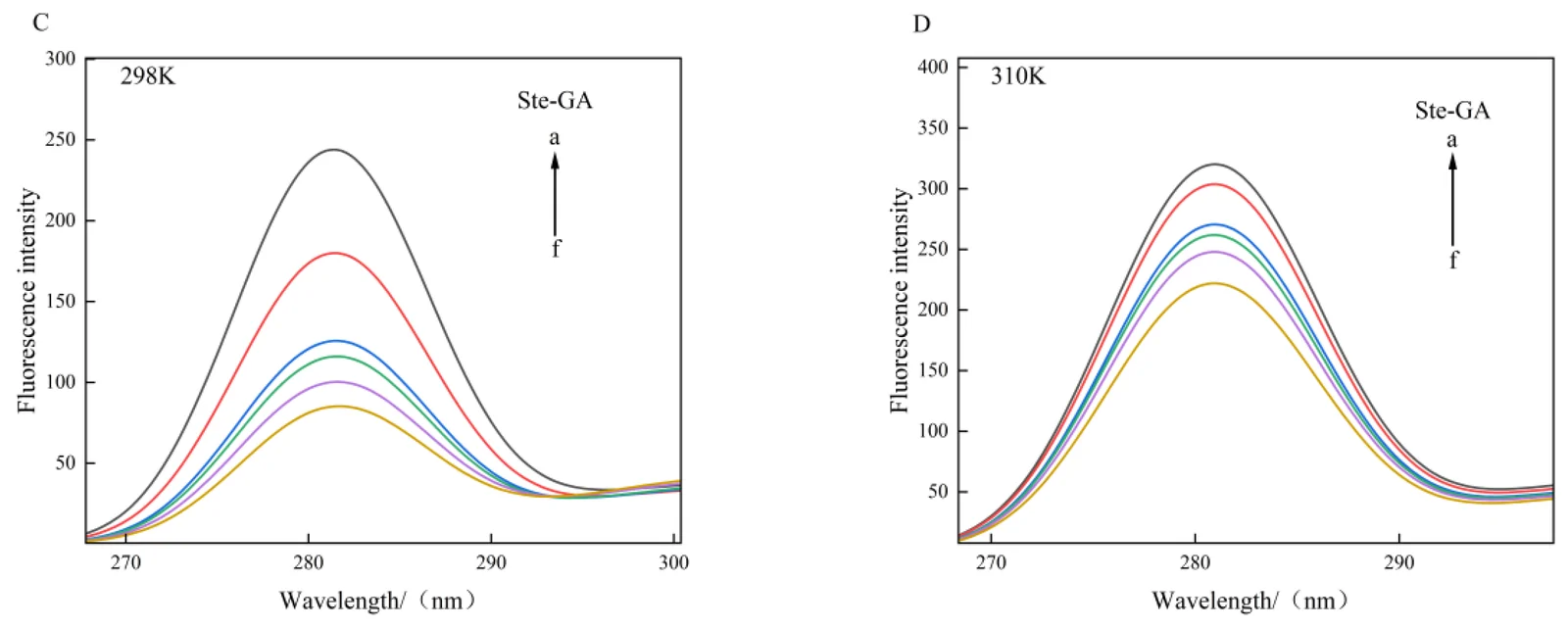
Fluorescence quenching spectra of Xyl, Ste and GA at 298 K (**A**,**C**) and 310 K (**B**,**D**). Note: (**A**,**B**) curve a–f corresponding to the concentration of xylitol added was 0.05, 0.1, 0.2, 0.3, 0.4, 0.5 mol/L; (**C**,**D**) curve a–f corresponded to stearic acid concentration of 0, 2.81 × 10^−3^, 5.62 × 10^−3^, 11.24 × 10^−3^, 16.86 × 10^−3^, 22.48 × 10^−3^ mol/L.

**Figure 3:**
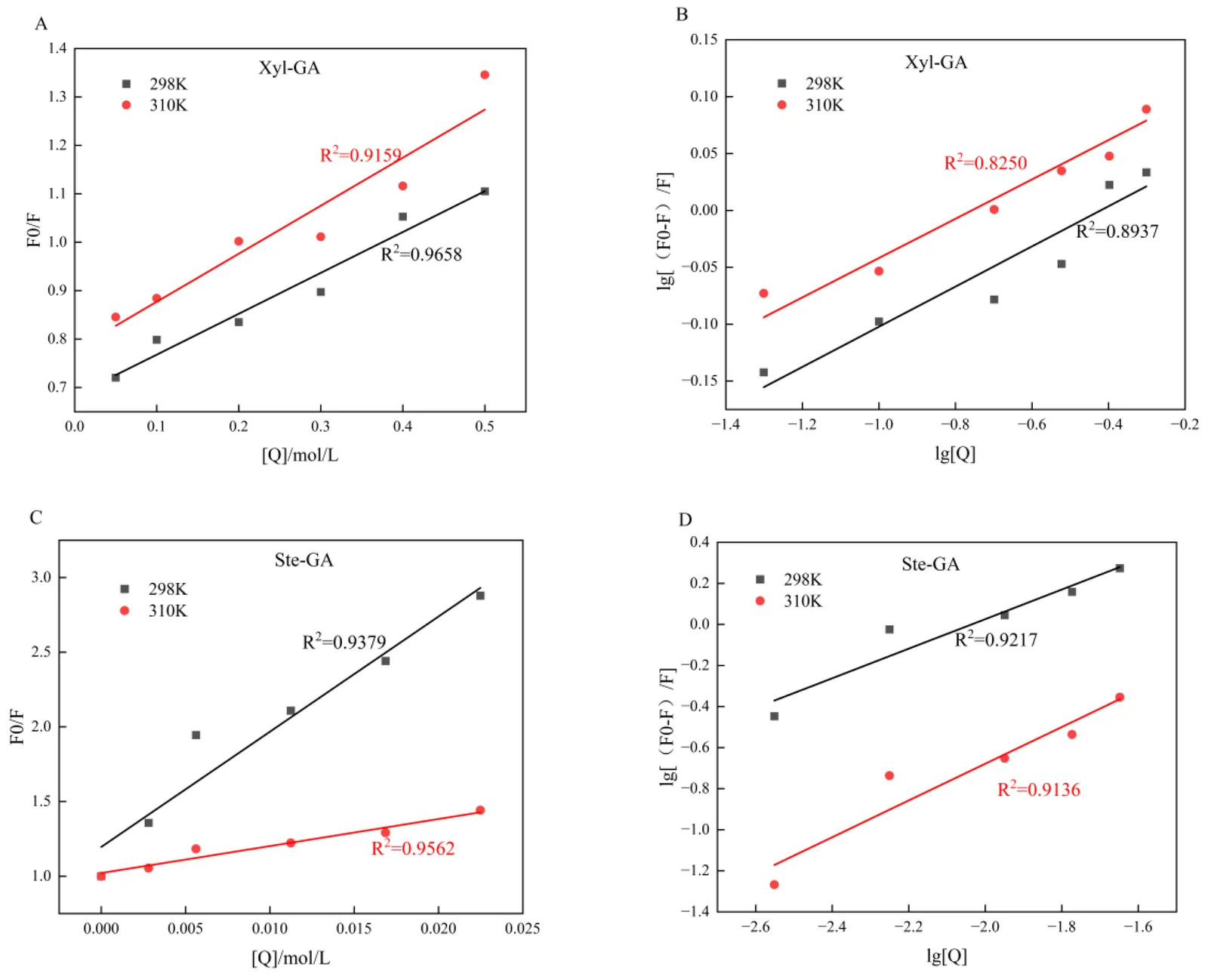
Quenching Stern-Volmer equation fitting diagram (**A**,**C**) and double logarithmic curve diagram (**B**,**D**) of the interaction between Xyl, Ste and GA.

**Figure 4:**
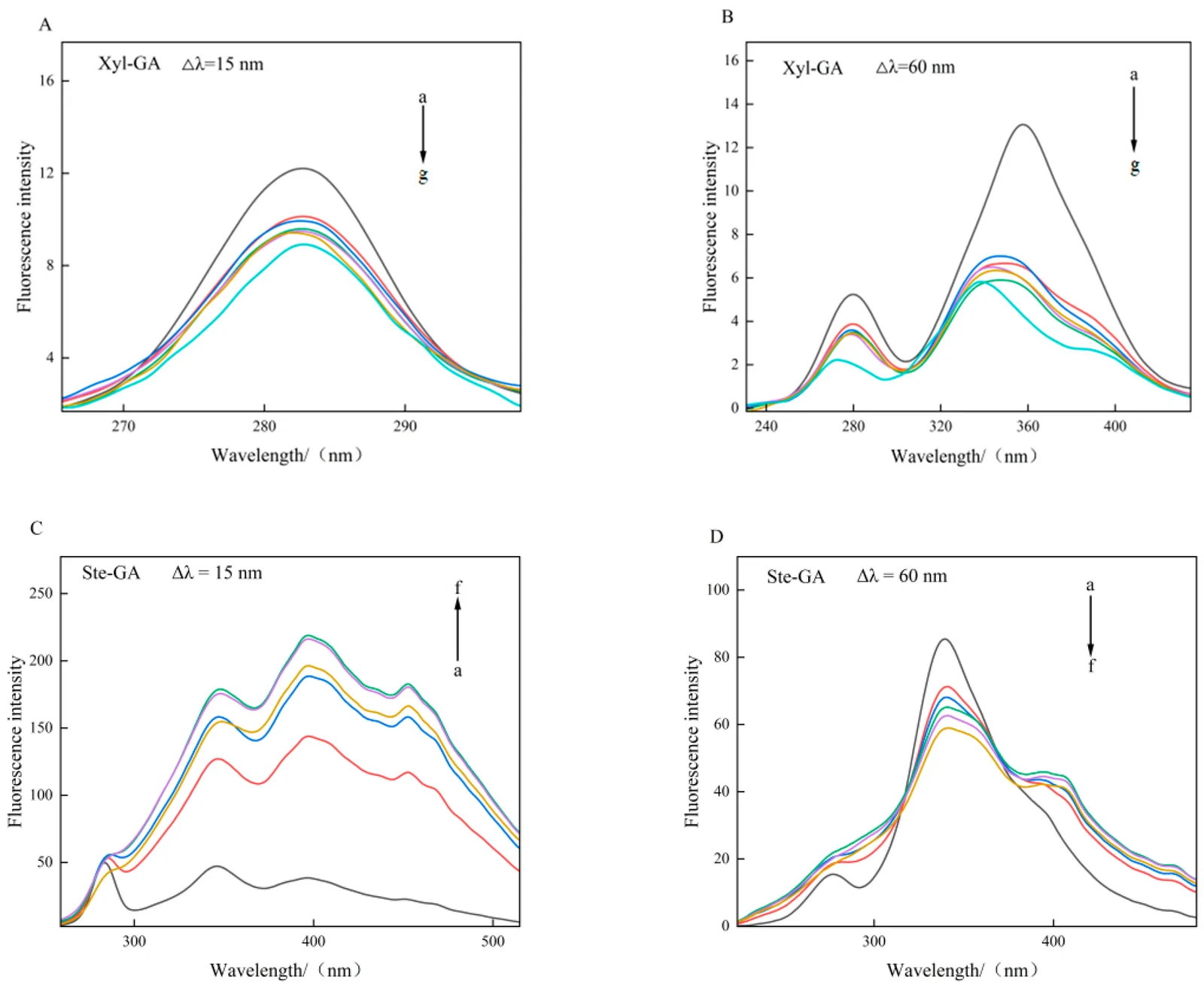
Synchronous fluorescence spectra of Xyl, Ste and GA at ∆λ = 15 nm (**A**,**C**) and ∆λ = 60 nm (**B**,**D**). Note: (**A**,**B**) curve a–g corresponding to the concentration of xylitol added was 0, 0.05, 0.1, 0.2, 0.3, 0.4, 0.5 mol/L; (**C**,**D**) curve a–f corresponded to stearic acid concentration of 0, 2.81 × 10^−3^, 5.62 × 10^−3^, 11.24 × 10^−3^, 16.86 × 10^−3^, 22.48 × 10^−3^ mol/L.

**Figure 5:**
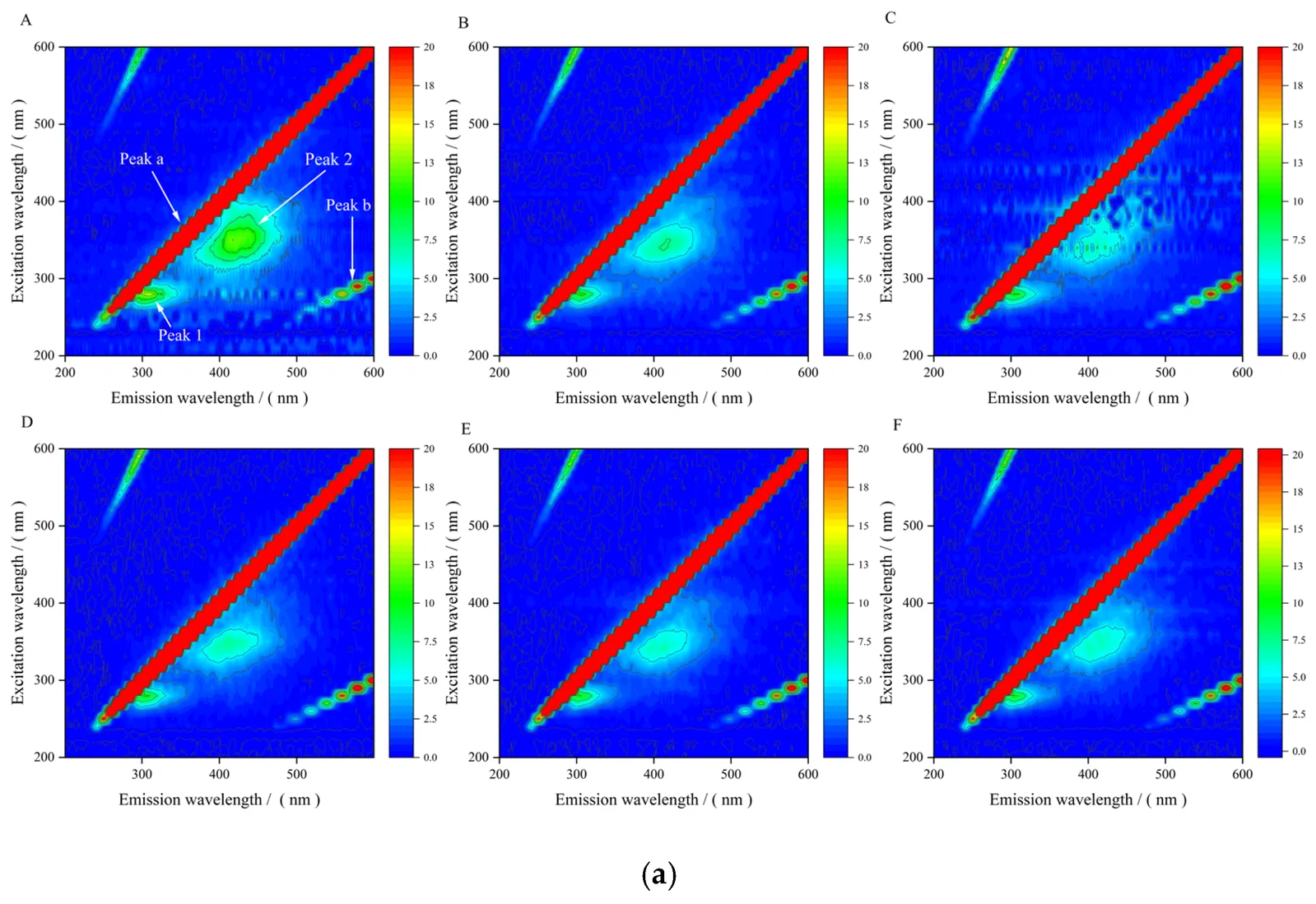

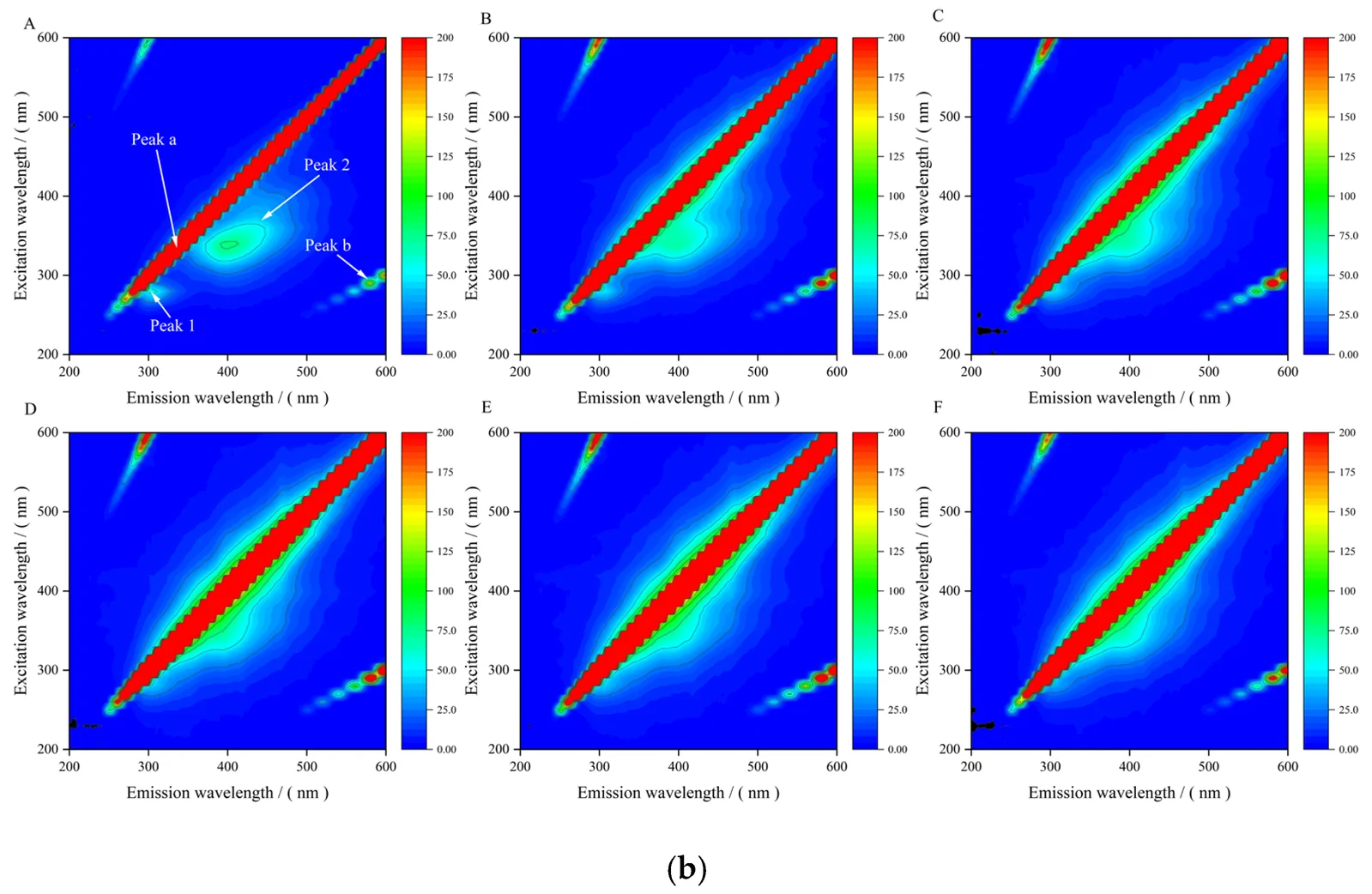
Three-dimensional fluorescence spectra of Xyl-GA and Ste-GA. Note: In (**a**), A–F represents the concentration of Xyl added at 0.05, 0.1, 0.2, 0.3, 0.4, 0.5 mol/L, respectively; in (**b**), A–F represent Ste concentration of 0, 2.81 × 10^−3^, 5.62 × 10^−3^, 11.24 × 10^−3^, 16.86 × 10^−3^, 22.48 × 10^−3^ mol/L, respectively.

**Figure 6:**
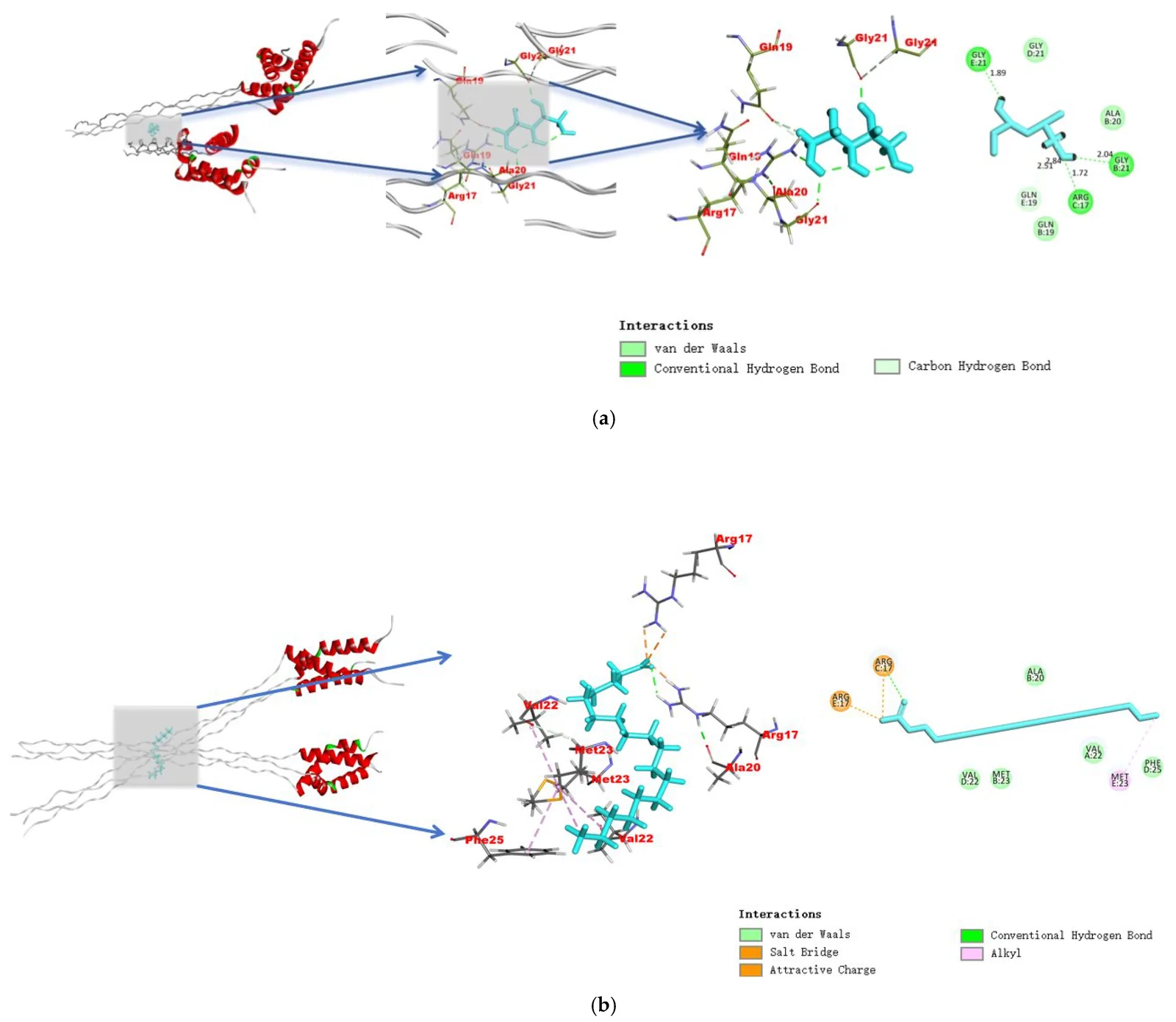
Molecular docking results of Xyl (**a**), Ste (**b**) and GA interaction.

**Table 1:** Preparation of stearic acid-gelatin interaction solution.

Serial Number	Gelatin Solution Volume/mL	Stearic Acid Solution Volume/mL	Deionized Water Volume/mL
1	5.0	0.0	5.0
2	5.0	0.5	4.5
3	5.0	1.0	4.0
4	5.0	2.0	3.0
5	5.0	3.0	2.0
6	5.0	4.0	1.0
7	5.0	5.0	0.0

**Table 2:** Binding constants and binding sites of Xyl, Ste and GA at 298 K and 310 K.

Specimen	Temperature T/K	Double Logarithmic Equation	ka/L/mol	n	R^2^
Xyl-GA	298	lg[(F_0_ − F)/F] = 0.1766 lg[Q] + 0.0743	1.1866	0.1766	0.8937
	310	lg[(F_0_ − F)/F] = 0.1729 lg[Q] + 0.1310	1.3521	0.1729	0.8250
Ste-GA	298	lg[(F_0_ − F)/F] = 0.7215 lg[Q] + 1.4696	29.4849	0.7215	0.9217
	310	lg[(F_0_ − F)/F] = 0.8945 lg[Q] + 1.1105	12.8973	0.8945	0.9136

**Table 3:** Thermodynamic parameters and interaction types of Xyl, Ste and GA at 298 K and 310 K.

Specimen	Temperature T/K	ΔG /kJ·mol^−1^	ΔH /kJ·mol^−1^	ΔS /kJ·mol^−1^K^−1^	Sort of Binding Force
Xyl-GA	298	−423.87	8356.14	29.46	hydrophobic interaction
	310	−777.43			
Ste-GA	298	−8383.80	−52.92	−149.46	Hydrogen bond, van der Waals force
	310	−6590.31			

For the same interaction, ΔH and ΔS remain constant with increasing temperature, while only ΔG varies.

## Data Availability

The original contributions presented in this study are included in the article. Further inquiries can be directed to the corresponding author.
